# Automated Skeletal Bone Age Assessment with Two-Stage Convolutional Transformer Network Based on X-ray Images

**DOI:** 10.3390/diagnostics13111837

**Published:** 2023-05-24

**Authors:** Xiongwei Mao, Qinglei Hui, Siyu Zhu, Wending Du, Chenhui Qiu, Xiaoping Ouyang, Dexing Kong

**Affiliations:** 1 Department of Radiology, Zhejiang University Hospital, Zhejiang University, Hangzhou 310027, China; xiongwei77@zju.edu.cn; 2 Department of Radiology, Zhejiang University Hospital District, The Second Affiliated Hospital, Zhejiang University School of Medicine, Hangzhou 310009, China; 3 School of Mathematical Sciences, Zhejiang University, Hangzhou 310027, China; qlhui@zju.edu.cn (Q.H.); qiugongsun@zju.edu.cn (C.Q.); 4 Zhejiang Qiushi Institute for Mathematical Medicine, Hangzhou 311121, China; siyu.zhu643@duke.edu (S.Z.); wending.du@duke.edu (W.D.); 5 School of Mechanical Engineering, Zhejiang University, Hangzhou 310027, China; ouyangxp@zju.edu.cn

**Keywords:** bone age assessment, transformer, deep learning, pediatrics

## Abstract

Human skeletal development is continuous and staged, and different stages have various morphological characteristics. Therefore, bone age assessment (BAA) can accurately reflect the individual’s growth and development level and maturity. Clinical BAA is time consuming, highly subjective, and lacks consistency. Deep learning has made considerable progress in BAA in recent years by effectively extracting deep features. Most studies use neural networks to extract global information from input images. However, clinical radiologists are highly concerned about the ossification degree in some specific regions of the hand bones. This paper proposes a two-stage convolutional transformer network to improve the accuracy of BAA. Combined with object detection and transformer, the first stage mimics the bone age reading process of the pediatrician, extracts the hand bone region of interest (ROI) in real time using YOLOv5, and proposes hand bone posture alignment. In addition, the previous information encoding of biological sex is integrated into the feature map to replace the position token in the transformer. The second stage extracts features within the ROI by window attention, interacts between different ROIs by shifting the window attention to extract hidden feature information, and penalizes the evaluation results using a hybrid loss function to ensure its stability and accuracy. The proposed method is evaluated on the data from the Pediatric Bone Age Challenge organized by the Radiological Society of North America (RSNA). The experimental results show that the proposed method achieves a mean absolute error (MAE) of 6.22 and 4.585 months on the validation and testing sets, respectively, and the cumulative accuracy within 6 and 12 months reach 71% and 96%, respectively, which is comparable to the state of the art, markedly reducing the clinical workload and realizing rapid, automatic, and high-precision assessment.

## 1. Introduction

In the medical field, human growth and development are mainly measured by ’age’, which can be defined by chronological age and biological age. The chronological age is determined by the date of birth. However, the actual growth and development of different individuals in the same age group may considerably vary due to different growth environments and nutritional levels. In particular, children or adolescents have considerable differences in their biological maturity. In contrast, biological age is an age inferred from the normal physiological and anatomical states of human body development and is an objective expression of physical maturity. Therefore, assessing the maturity of the human body by measuring biological age is necessary. Bone age was first used in medical pediatrics. It not only determines the biological age of children but also helps understand the growth and development potential of children as well as the trend of sexual maturity. Abnormal bone age is a symptom of some pediatric endocrine diseases. Therefore, BAA is considerably useful for the diagnosis of some pediatric endocrine diseases and provides timely and effective treatment for patients with abnormal growth [1]. In addition, bone age can provide scientific and objective biological age detection and is often used in the identification of sports athletes [2] and judicial decision [3,4].

The traditional BAA process usually involves pediatric radiologists’ observation of the maturity of the phalanges, carpal bones, radial, and ulnar based on left hand X-ray images. These images are then compared with established criteria to determine the bone age of patients. The main clinical methods used to assess bone age are the Greulich–Pyle atlas (GP) [5] and the Tanner–Whitehouse scores (TW) [6,7,8]. The GP method is a series of standardized radiographic atlas based on growth studies in children. Bone age is assessed by directly comparing the X-ray images of the subject with the standard atlas. GP has been widely practiced internationally due to its simplicity, clarity, and ease of use. However, GP is limited by its high subjectiveness and uncertain accuracy. The TW method scores the maturity of specific epiphysis, and the skeletal age is derived by checking the bone age scale (currently revised to TW3). This method is more objective and more robust than GP. However, the TW assessment process is complex and requires a certain amount of time for an experienced pediatric radiologist to complete the BAA. Therefore, many researchers have worked to develop fast, accurate, and highly objective methods for BAA.

Two types of techniques are available in computerized BAA: traditional and deep learning (which are described in Section 2). Most traditional techniques require manual feature selection, with the disadvantages of large errors, low stability, and no real automated evaluation. Deep learning-based methods use convolutional neural networks (CNNs), which can automatically extract image features and then derive the age of bones by regression or classification. These methods are faster, more stable, and more accurate than previous methods. Some methods for BAA have produced promising results; however, the field still faces the following problems. (1) This assessment is a time-consuming and labor-intensive task; it is highly dependent on the experience and operation of physicians. Even two evaluations performed by the same physician may have different results, and robust evaluation results cannot be obtained. (2) The quality of X-ray images is variable due to the different equipment, methods, and standards used for taking hand bone radiographs. Moreover, the presence of background noise in the images can interfere with the accuracy of the BAA. (3) Compared with other large scale datasets, the hand bone X-ray dataset is still slightly insufficient, which prevents the model from learning comprehensive features, affecting the generalization performance of the model. (4) Deep learning methods have low ROI attention on hand bone images but still achieve promising results. The prediction performance can still be further improved by adding previous knowledge to the deep learning framework.

The transformer method [9] was first used in natural language processing and has become popular in the field of computer vision [10,11] in recent years. This approach mainly aims to apply a self-attentive layer on the input sequence to capture the relationship between local regions. The transformer segments the image into different patches. The TW3 score method focuses on the 18 key joints and bones rather than the entire hand image. Conceptually, similarities are observed between the two methods. Therefore, the swin transformer is introduced into the feature extraction module. In addition, the transformer considers the position encoding information (position token). This phenomenon inspired the innovative encoding of image sex information (sex token). In this paper, a new automatic BAA system is proposed. Firstly, an object detection network is used to quickly localize the local patches. Then, the ROI features extracted by CNN are fused with the sex token information. Finally, a transformer is used to capture the information among ROIs to predict bone age. A new hybrid loss function is used to penalize the difference between the prediction results and the true label. The contributions of this paper are as follows:

1. Inspired by the reading process of pediatric radiologists, object detection is applied to BAA, using YOLOv5 real time efficient detection. The detected ROIs are the specific regions mentioned in the TW3 guidelines. Furthermore, automated hand bone pose alignment is proposed to reduce the impact of hand bone mirroring, flipping, and rotation caused by the clinical photography in the original dataset.
2. Sex is a piece of known information, in this study, the biological sex label is used as the input to the network; that is, the sex token is fused with the obtained features by convolution. Different from the summation method used by the transformer for location encoded information, the multiplication method is used to fuse the features.
3. A new hybrid loss is used in this study. The mean loss is used to penalize the distribution between the mean of the estimated and labeled ages to ensure the accuracy of the assessment, while the variance loss is used to penalize the variance of the distribution of the estimated age values to ensure the robustness of the assessment. The cross-entropy loss function guarantees the convergence of the networks.

The ROIs features of the TW scores method are fully explored to further improve accuracy, which can help to simplify the workflow, reduce the inter-operator variation, and provide a reference for clinical diagnosis. The rest of the paper is organized as follows. Section 2 briefly summarizes the BAA methods based on traditional techniques and deep learning. Section 3 comprehensively describes the proposed model. Section 4 presents the experiments, and Section 5 provides further discussion.

## 2. Related Work

Manual assessment in the clinical GP and TW methods is unstable, unreliable, and time-consuming. Therefore, scholars have begun to explore the use of computers to share the work of reading and analysis, and a considerable amount of exploratory work in computerized BAA systems has been performed. However, no corresponding intelligent system has been widely promoted and applied in clinical practice until now. This section briefly introduces the relatively representative work in the development of computer-aided systems for BAA.

### 2.1. Computerized BAA Methods Based on Conventional Techniques

Such methods use traditional image processing algorithms for processing and generally require manual definition of features. Michael et al. [12] developed the ’HANDX’ hand bone measurement system, which uses an adaptive contour approximation algorithm to approximate the contours of the bones and measure the length and width of each bone contour. The system disregarded image noise and anatomical differences, reducing the sensitivity to different subjects, but the system requires previous acquisition of the hand position for model construction. Niemeijer et al. [13] proposed an automated BAA active shape model with skeletal shape and texture features. The active shape model was used to detect the location and shape of the region of interest in the image to be measured. The correlation coefficient was calculated by comparing it with the average image of the ROI in the TW criterion. The developmental grade corresponding to the highest value of the correlation coefficient was selected as the skeletal age. Hsieh et al. [14] introduced an automated BAA system for image geometric feature analysis. The ROI features of the finger and wrist bones were first extracted, and a back propagation algorithm, radial basis function, and support vector machine neural network were then used to assess the bone age. Thodberg et al. [15] designed and developed a fully automated BAA system called ’BoneXpert’. First, this system reconstructed the edges of the bone masses, calculated the bone age of each bone mass individually, and finally converted the bone age of each bone mass to the overall bone age. The system was fully automated and demonstrated a high degree of accuracy. However, inputting the relationship between the skeletal and chronological ages of the target race is necessary during application. In addition, the system was weak to abnormal X-ray images. Sheshasaayee et al. [16] proposed another model for BAA based on a dictionary learning algorithm, which used a noise level estimation algorithm based on principal component analysis to reduce noise in X-ray images, classified the images by the Kernel Support Vector Machine algorithm to obtain the similarity scores of the images to be tested, and derived the prediction of bone age using a dictionary learning algorithm with recognition power.

### 2.2. Deep-Learning-Based Computerized BAA Method

Deep learning has a powerful learning capability to extract more feature information than traditional image processing methods, and BAA can be regarded as a regression or classification problem. Štern et al. [17] first proposed a deep CNN-based method for BAA with an MRI of the hand, combined with the ossification stage of 13 bones, to predict the skeletal age. Spampinato et al. [18] used deep learning methods to assess the bone age from X-ray images. After testing several CNNs on public data, they found that the average error of bone age was 9.6 months, providing a direction for BAA in deep learning. Lee et al. [19] at Harvard Medical School proposed a fully automated BAA model based on transfer learning with CNNs, which first segmented the palm region from the background using CNNs and then utilized the full map as input to assess the bone age with pretrained GoogLeNet. The results were coarse-grained estimates based on integers because the system was evaluated on the basis of the full graph. Iglovikov et al. [20] designed several network structures for BAA. First, the palm was segmented using a network called U-Net. Three key points were then detected by VGG-Net to eliminate the interference from palm deformation and rotation. According to the two different tasks of regression and classification, two network structures were designed with the full image as input. An MAE of 4.97 months was achieved by integrating the two networks. Wu et al. [21] proposed a residual attention-based network for the hand BAA. First, a Mask-RCNN subnetwork was used to segment the hand regions to reduce the influence of the irrelevant regions on the model. A residual attention network was then used to focus on the key components in the images to finally predict the skeletal age. The MAE of this method was 7.38 months. María et al. [22] introduced the radioactive hand pose estimation dataset with hand detection and hand pose estimation as new extraction tasks, combining fine-grained ROI and local analysis to train the network on both datasets. The presence of low-quality X-ray images is inevitable in real medical scenarios. Guo et al. [23] proposed a regression model, namely BoNet+, based on DenseNet, which was developed to estimate the bone age accurately with poor image quality. Tentative proposals also indicated that if the expressiveness of the CNN model was sufficiently high, then one model could handle multiple tasks simultaneously. Unlike hand bone X-ray images, Aydin et al. [24] collected knee X-ray images to predict the bone age and evaluated these images in independent and external validation cohorts, demonstrating the feasibility of automated knee X-ray age assessment. This work provided a valuable reference for further evaluation of bone age in children.

Numerous BAA methods that use MRI and ultrasound images are available. MRI has no ionizing radiation and has high image resolution but can display the normal and pathological features of bone and cartilage [25]. Tomei et al. [26] showed a strong correlation between the MRI assessment of bone and chronological ages and demonstrated cartilage maturation, suggesting that the MRI assessment of bone age may be more accurate than traditional X-ray images. Widek et al. [27] used the GP atlas method to grade the MRI, and the statistical data processing was consistent with the X-ray examination, which provided a new reference value for BAA. As a noninvasive means of examination, ultrasound has rarely been used for bone age in the past. Some progress in BAA has been observed in recent years. Wan et al. [28] discussed the diagnostic effect of ultrasound in assessing abnormal bone age in children. With radiographic bone age as the reference standard, the paired sample *t*-test was used to determine the statistically significant difference between groups. The established ultrasonic bone age system provides a new idea for BAA.

From the above studies, traditional BAA methods have a large human factor and are highly subjective. Therefore, these methods have the disadvantages of being tedious and containing large errors. Traditional image processing methods, which have weak learning capability, poor robustness, and no automated assessment, must perform manual feature selection. Deep learning-based BAA method uses CNNs, which can automatically extract image features and then derive the bone age by regression or classification, demonstrating faster, more stable, and more accurate results than previous methods.

## 3. Methodology

**Figure 1:**
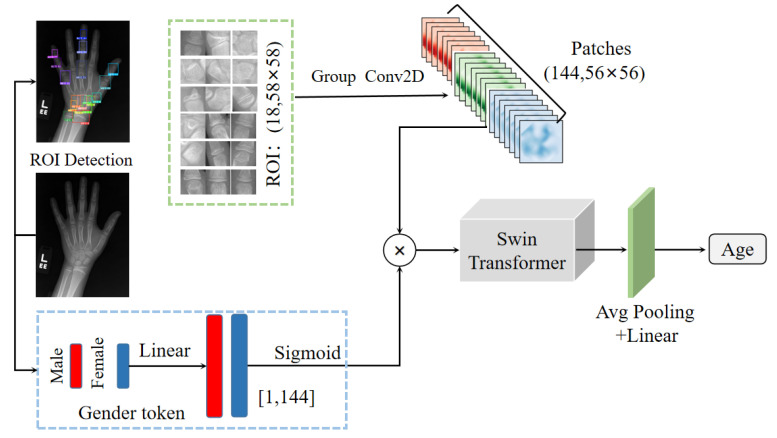
Overall process framework.

This section comprehensively describes the architecture of the automated BAA network in this study. This architecture comprises two phases, ROI detection and swin transformer bone age prediction. Figure 1 shows the basic network architecture of the BAA. Each phase is described in more detail in the following sections.

### 3.1. ROI Detection Using YOLOv5

The method of TW3 scores disregards the overall hand features but focuses on the maturity of the 18 key joints and bones. This subsection identifies the 18 ROIs by object detection based on the TW3 method.

**Figure 2:**
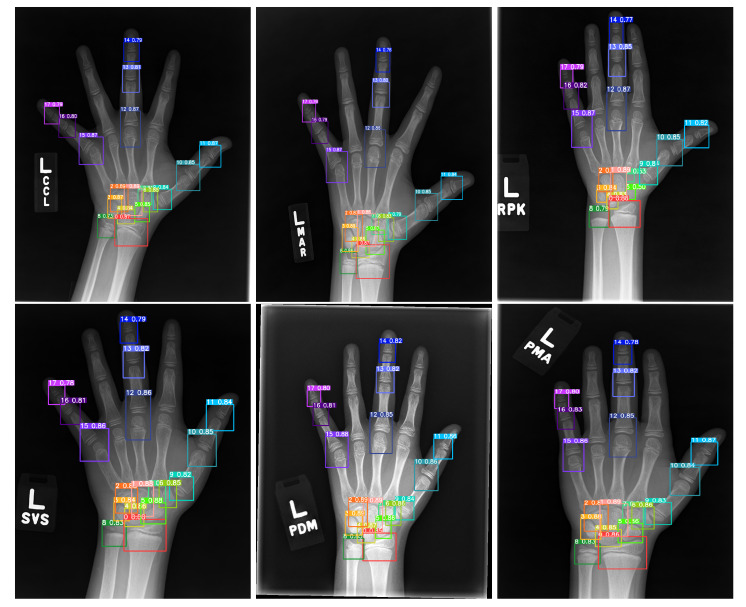
ROI detection results.

Object detection is a major direction in computer vision. Deep-learning-based object detection algorithms can be divided into two major categories. The first category is a two-step detection network, which first generates candidate regions, followed by classification and location refinement. This class is represented by the R-CNN algorithm [29,30,31]. The second category is a one-step detection network, which directly generates the class and location coordinate values of objects. Typical algorithms include YOLO [32,33,34] and SSD algorithms [35]. YOLO detection is fast and can be detected in real time. In this study, the YOLOv5 algorithm was used, and the detection results are shown in Figure 2.

### 3.2. Hand Bone Alignment

Due to the clinical X-ray shooting angle and human operation, the angular deviation of each hand bone posture was large, with mirror image of left and right hand bones, up and down flip, and rotation scaling. The left hand bone was adjusted to a uniform posture (palm face down, middle finger in the middle, and in a vertical position) to reduce the difficulty of feature extraction in the later automatic BAA model.

The specific alignment operation first discriminated the data of left–right mirroring and up–down flipping by the detection results. This operation then executed the inverse operation to facilitate the return of the image to the left hand pose and the operation of rotation standard alignment. Then, 18 ROIs were segmented after the pose correction.

For left–right mirroring, the left–right mirroring alignment was performed by determining the mean value of the centroid longitudinal coordinates of the 8th, 15th, 16th, and 17th ROIs and the 0th, 9th, 10th, and 11th ROIs. Specifically, if $Y_{\mathrm{left}}>Y_{\mathrm{right}}$, then the image was mirrored left and right to flip, where $Y_{\mathrm{left}}$ denotes the mean value of the vertical coordinates of the 8th, 15th, 16th, and 17th ROIs, and $Y_{\mathrm{right}}$ denotes the mean value of the vertical coordinates of the 0th, 9th, 10th, and 11th ROIs.

For up–down flip, a 180° rotation alignment was performed by determining the mean value of the horizontal coordinates of the center points of the 0th, 8th, 1st, and 2nd ROIs and the 11th, 13th, 14th, and 17th ROIs. Specifically, if $Y_{\mathrm{up}}>Y_{\mathrm{down}}$, then the image was flipped up and down, where $Y_{\mathrm{up}}$ denotes the mean value of the cross coordinates of the 11th, 13th, 14th, and 17th ROIs, and $Y_{\mathrm{down}}$ denotes the mean value of the cross coordinates of the 0th, 8th, 1st, and 2nd ROIs.

**Figure 3:**
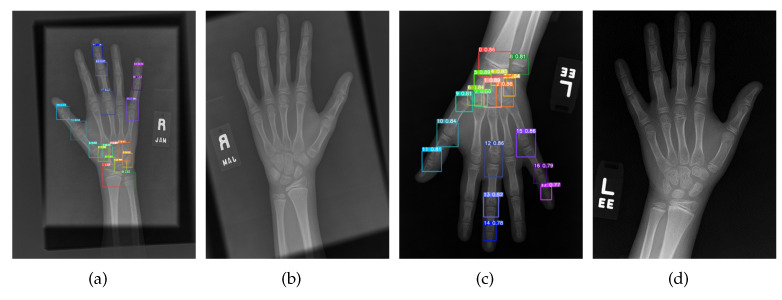
Image alignment. (**a**) Mirror, (**b**) mirror alignment, (**c**) flip, (**d**) flip alignment.

For rotation, the center point coordinates of the 1st and 14th ROI were aligned to the hand bone by detecting that the middle finger was in a vertical position. The angle between the line of the two coordinates and the Y direction of the image, which is the angle of rotation, was calculated. Figure 3 provides an example of mirroring and flipping alignment.

### 3.3. Transformer Framework

Biological sex is key priori information in BAA. The sex label is also used as an input to the network. The cropped ROIs obtained were reconstructed into a 3D matrix of size (144,58×58). Similar to the position-encoded information of the image in the transformer (called the position token), the sex label corresponding to the bone age image, namely the sex token, was encoded and multiplied and then fused with the ROI patches as the input of the swin transformer after sigmoid activation. Specifically, the biological sex information was a two-dimensional vector; that is, (0,1) and (1,0) represent males and females, respectively. After a linear layer, softmax was utilized to transform its dimension into [1,144], and a dot product operation was then performed with the ROI feature map. Therefore, each point in the feature map contained sex information.

**Figure 4:**
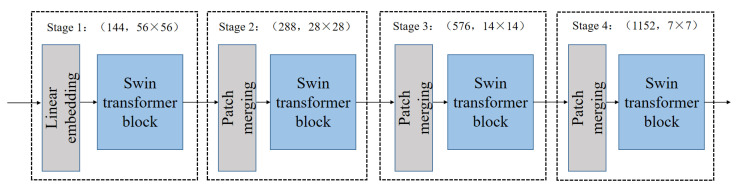
Swin transformer architecture.

The structure of the transformer framework, which is similar to a convolutional hierarchical structure, is shown in Figure 4. The resolution of each layer becomes half of the original, while the number of channels doubles. The ROI features of the input hand bone were used to construct feature maps of different sizes in four stages, with each stage comprising the following two parts: image block merging (the first block is a linear embedding layer) and the swin transformer block. The ROI features utilized the shift window multihead self-attention (SW–MSA) to interact with various ROI feature maps, while the features within the ROI used the window multihead self-attention (W–MSA).

**Figure 5:**
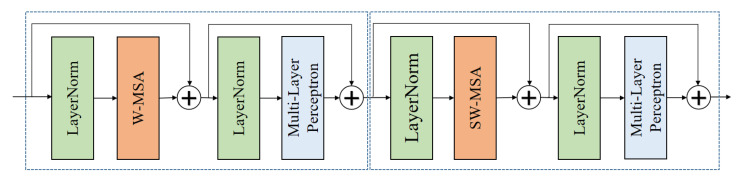
Swin transformer blocks.

The swin block is shown in Figure 5, where the first block first involves the feature map passing through the LayerNorm, W–MSA, and then performing skip connections. The map then passes through the LayerNorm and multilayer perceptron again and then performs skip connections. The second block repeats the above steps, but the difference lies in the switching to the SW–MSA.

### 3.4. Hybrid Loss Function

A new hybrid loss function, which contains three parts of loss, the mean–variance–cross-entropy loss function, is presented in this study. Specifically, we let $x_{i}$ represent the *i*-th sample of the feature vector and $y_{i}\in \left(1,2,\ldots ,K\right)$ represent the corresponding truth age label, $f\left(x_{i}\right)\in R^{N\times M}$ indicates the output of the network before the final fully connected layer, and $\theta \in R^{K\times M}$ represents the parameters of the final fully connected layer. The final output of the fully connected layer can then be expressed as $z=f\left(x_{i}\theta^{T}\right)\in R^{N\times K}$.

The softmax probability can be calculated using the following equation:

(1) $$p_{i,j}=\frac{\exp(z_{i,j})}{\sum_{k=1}^{K}\exp\left(z_{i,k}\right)},$$

where j∈(1,2,…,K) is the class label, $p_{i}$ indicates the age distribution of the estimated sample *i* in all *K* classes, and $p_{i,j}$ indicates the probability that sample *i* belongs to class *j*. The mean $m_{i}$ and variance $v_{i}$ can be calculated as follows:

(2) $$m_{i}=\sum_{j=1}^{K}j*p_{i,j},$$

(3) $$v_{i}=\sum_{j=1}^{K}p_{i,j}*{\left(j-m_{i}\right)}^{2}.$$

The mean loss penalizes the difference between the mean $m_{i}$ of the estimated age distribution and the true age. The mean loss can be calculated in accordance with Equation (1) as shown below.

(4) $$L_{m}=\frac{1}{2N}\sum_{i=1}^{N}{\left(m_{i}-y_{i}\right)}^{2}=\frac{1}{2N}\sum_{i=1}^{N}(\sum_{j=1}^{K}j*p_{i,j}-y_{i}{)}^{2},$$

where *N* is the batch size. Different from the softmax loss that focuses on the classification task, the mean loss emphasizes the regression task, where the $L_{2}$ is used to measure the distance between the mean of the estimated age distribution and the ground truth age. Thus, it complements the softmax loss.

The dispersion of the estimated age distribution is penalized for the variance loss. The variance loss can then be calculated on the basis of Equations (2) and (3) as follows:

(5) $$L_{v}=\frac{1}{N}\sum_{i=1}^{N}v_{i}=\frac{1}{N}\sum_{i=1}^{N}\sum_{j=1}^{K}p_{i,j}*{\left(j-\sum_{k=1}^{K}k*p_{i,k}\right)}^{2}.$$

This variance loss requires the concentration of the estimated distribution on a small range of means. Using a Gaussian distribution as an example, the variance loss increases the sharpness of the distribution. Obtaining accurate age estimates with small confidence intervals but high confidence is helpful. However, the mean variance loss may markedly fluctuate in the early training stage because it is only used at the end of the randomly initialized network, so cross entropy is jointly used to help the network converge as early as possible. The final loss function is as follows:

(6) $$L=L_{s}+\lambda_{1}L_{m}+\lambda_{2}L_{v}=\frac{1}{N}\sum_{i=1}^{N}-\log p_{i,y_{i}}+\frac{\lambda_{1}}{2}{\left(m_{i}-y_{i}\right)}^{2}+\lambda_{2}v_{i},$$

where $\lambda_{1}$ and $\lambda_{2}$ are the two hyperparameters that balance the effects of the subloss function in the joint loss.

## 4. Experimental Results

This section presents extensive experiments to demonstrate the performance of the two-stage convolutional transformer network. The subsections below present details of the dataset and preprocessing, network training, experimental results, and comparisons with other BAAs.

### 4.1. Dataset and Preprocessing

The experimental data were obtained from the 2017 Pediatric Bone Age Challenge organized by the RNSA, which has a total of 14,236 X-ray images (12,611 in the training set, 1425 in the validation set, and 200 in the testing set).

**Figure 6:**
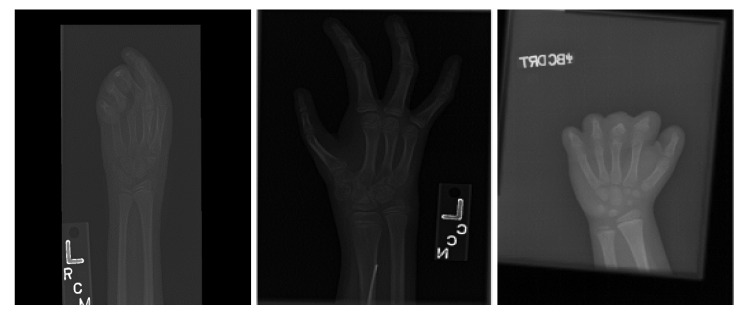
Example of abnormal images.

Data cleaning phase: Images with ROI category numbers less than 14 detected in the training set were removed in this study. This set excluded a portion of images with dysplasia or lesions, as shown in Figure 6. The final number of samples in the training set was 12,574.

### 4.2. Network Training

For the ROI detection network, 200 images were selected as the training set and 100 as the testing set in proportion to age; these images were manually labeled, trained, and tested with YOLOv5. A total of 100 images was selected at one time from the remaining unlabeled images during testing. We observed the results and added the images with a wrong ROI number less than or equal to 2 to the training set by modifying them and retraining them. The average time spent on modifying each image was less than 10 s. The above process was repeated until the effect of the testing set met the experimental requirements. The above process was iterated eight times in total, and 526 images were labeled in total. The detection precision, recall, and F1 scores of the testing set were 0.998, 0.997, and 0.996, respectively, and the values of the mAP_0.5 and mAP_0.5:0.95 were 0.995 and 0.676, respectively.

For the ROI detection network, the batch size, IoU threshold, and epoch were set to 64, 0.5, and 100, respectively, with an initial learning rate of 0.01 and three sizes of anchors, [10,13,16,30,33,23], [30,61,62,45,59,119], [116,90,156,198,373,326], respectively. For the evaluation network, Adam was chosen as the optimizer with an initial learning rate of 0.001, decaying by 1/3 every 10 cycles, and two hyperparameters $\lambda_{1}=0.001$ and $\lambda_{2}=0.0002$ in the loss function.

### 4.3. Results

This work used the MAE and root mean square error (RMSE) for quantitative assessment, which are calculated as follows:

(7) $$\mathrm{MAE}=\frac{1}{N}\sum_{i=1}^{n}\left|y_{i}-{\hat{y}}_{i}\right|,$$

(8) $$\mathrm{RMSE}=\sqrt{\frac{1}{N}\sum_{i=1}^{n}{\left|y_{i}-{\hat{y}}_{i}\right|}^{2}},$$

where $y_{i}$ and ${\hat{y}}_{i}$ indicate the predicted bone age and true bone age labels, respectively.

Table 1 shows the MAE and RMSE results by age group for the different groups. The model was effective in assessing the bone age with high accuracy for people of any sex and age range from 0 to 20 years.

**Figure 7:**
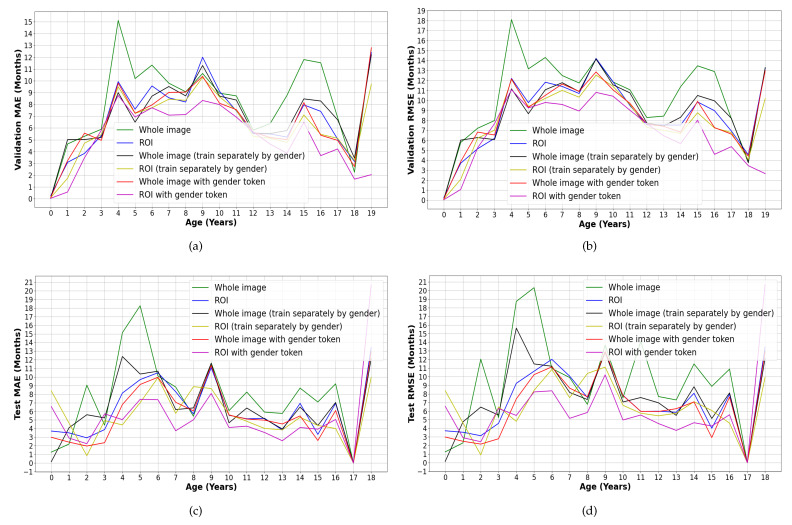
Comparison of the experimental results by age. (**a**) MAE of the validation set, (**b**) RMSE of the validated set, (**c**) MAE of the testing set, and (**d**) RMSE of the testing set.

The following ablation experiments were also performed to verify the validity of the ROI and sex token: whole image only; ROI only; whole image (trained separately by sex); ROI (trained separately by sex); whole image (trained with sex token); ROI (trained with sex token). The results are shown in Table 2, while the MAE and RMSE results for each age group are shown in Figure 7.

**Figure 8:**
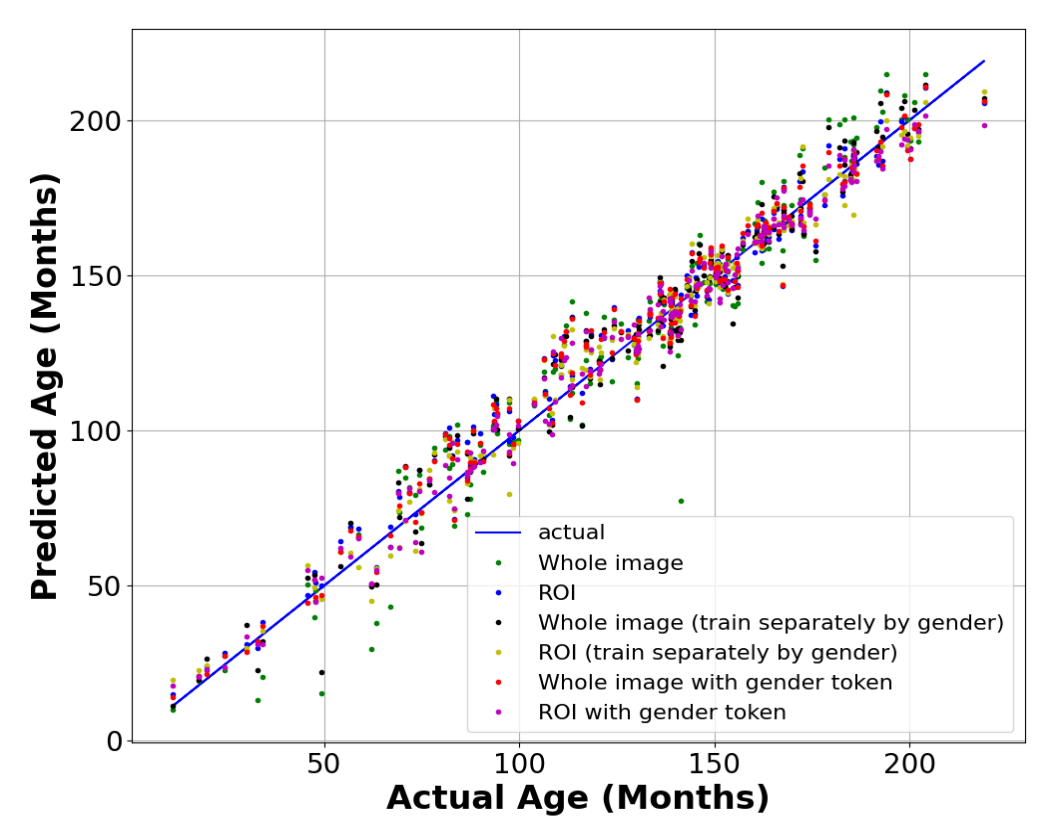
Bone age prediction identity line.

Figure 8 shows the identity lines of each model for predicting the bone age results on the testing set. The ROI with the sex token showed the best performance, demonstrating stability at all ages, and its mean value was closest to the true line.

**Figure 9:**
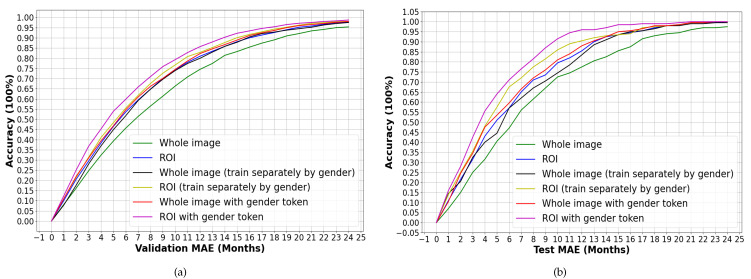
Cumulative accuracy curves. (**a**) Accuracy on the validation set, (**b**) accuracy on the testing set.

Moreover, the cumulative accuracy of each model (the error gradually increased from 0 months to 24 months) was calculated. A series of accuracy rates was obtained to generate cumulative accuracy curves, as shown in Figure 9. The horizontal axis is the error in month, while the vertical axis is the cumulative accuracy rate. In the validation set, an MAE less than or equal to 6 months and 12 months reached 60% and 86% accuracy rates, respectively. In the testing set, the accuracy rates of the MAE less than or equal to 6 months and 12 months reached 71% and 96%, respectively.

### 4.4. Comparison with Other Technologies

In the BAA, some methods were either tested on private datasets or their source code was unavailable; thus, their results were not reproducible or usable as baselines. For comparison purposes, methods that used the RSNA dataset for evaluation were selected (Table 3 shows the specific results).

## 5. Discussion

Notably, the results of the models in the testing set were significantly better than those in the validation set, as seen in Table 1 and Table 2. This finding is mainly due to the following two reasons: (1) In this challenge, there was a significant disparity between the training and testing data sets. The testing set had a smaller amount of data compared to the validation set, only 200 samples, which ineffectively represented the distribution of each age group. In addition, compared to the validation set, there were no samples of 17–18 and 19–20 years old in the testing set. (2) The image and labeling qualities of the testing set were higher than the validation set, and each image in the testing set was independently labeled and cross validated by three radiologists.

As can be seen from Table 2, extracting 18 ROIs led to better results compared to the images of the whole hand. The MAE obtained from the training based on the 18 ROIs without considering sex (6.22) was even lower than that obtained from training based on the whole hand with separated men and women (8.094). Therefore, the whole hand image contains numerous influences that do not correlate well with bone age, such as joints, muscles, and background. The process of extracting ROIs can exclude the interference of these areas with the bone age prediction. Meanwhile, experienced doctors can accurately assess the bone age through the 18 ROIs, which is consistent with the experimental results. Medically, the bone age considerably varies between males and females, and similar X-ray pictures of girls may be one to two years older than boys. Therefore, the sex is also important information when doctors diagnose bone age. Many existing BAA models also encode sex information into the model with excellent results.

The current experiments also demonstrated the importance of the sex token information. The model results on the validation and testing sets were improved after performing the sex token. The MAE based on the whole hand moved from 8.094 to 5.901 months, while for the ROI, it moved from 6.22 to 4.586 months. The effect of the sex token was better than sex separation training. The best results were obtained by generating a sex token with full concatenation and sigmoid activation and acting on the feature map of ROIs.

As shown in Table 3, Larson et al. [36] used ResNet–50 as a feature extractor to train and classify the probability distribution from 0 to 19 years. A mean error of six months was achieved in the RSNA testing set. González et al. [37] fused information from identity markers with visual features from raw hand bone X-rays and used this representation to estimate the relative bone age with an MAE of 5.47 months. Liu et al. [38] applied ranked learning for the BAA and used VGG–U–Net to segment the hand and wrist. A GAN network was then constructed to assess the bone age using a rank-monotonicity loss to improve the performance, yielding an MAE of 6.05 months. The top three of the RSNA Pediatric Bone Age Machine Learning Challenge [39] are discussed herein. First place used the Inception v3 architecture for pixel information and was connected to sex information. The connection demonstrated an additional dense layer to enable the network to learn the relationship between pixel and sex information. Second place used transfer learning and a finetuned ResNet–50 architecture pretrained on the ImageNet dataset. The model was trained using overlapping blocks of images, and the median of the patched predictions was selected as the prediction result. Third place developed a new variant of CNN by creating the Ice module. The dataset was divided into five parts, and a model was trained on each part, integrating the four best parts to form the final output.

Koitka et al. [40] simulated and accelerated the workflow of the TW scores method. The bone age was estimated from detection and regression networks that identified the ossified region and a regression network that identified the specific region and sex. The method provided self-explanatory results for radiologists. Liu et al. [41] proposed a single-stage attention recognition CNN. The attention module automatically discovered and extracted bone sites, while the recognition module learned features from the extracted bones and evaluated the bone age. In addition, the evaluation results were fed back to the attention module for optimizing bone extraction. The two modules reinforced each other, and the entire network could be trained end-to-end without human intervention. The obtained MAE was 4.38 months. Chen et al. [42] used an attention-guided approach to locate discriminative regions automatically for BAA without any additional annotation using joint age distribution learning and expectation regression to exploit the 339 ordered relationship between hand images of different individual ages, leading to additional robust bone age estimates with an MAE of 4.4 months.

In contrast to these works, this paper explored the application of object detection and transformer in automatic BAA. The BAA system can automatically estimate the skeletal maturity based on the proposed method. The accuracy of this method is similar to that of expert radiologists and is superior to most existing models.

## 6. Conclusions

A two-stage neural network with hybrid loss was proposed in this paper for BAA, which requires minimal time for diagnosis, reduces the workload of the clinician, and achieves high accuracy. Different from the existing BAA method based on CNN, the method first simulates the TW3 reading process of clinical experts and extracts 18 ROIs as the first step. These ROIs are the key local features in BAA, which overcome the shortcomings of the low ROI attention in existing deep-learning-based methods. Second, the alignment of hand bone posture helps the model identify and extract features, and the fusion of prior information on the sex improves the evaluation accuracy. The swin transformer SWA not only extracts feature information in the ROI area but also interacts with other ROIs to isolate multiscale features, which is similar to the TW scoring method that grades and aggregates ROIs individually. The hybrid loss strategy can effectively constrain the distribution of estimated ages, which increases the accuracy and stability of the age estimation. The experimental results show that the proposed method exhibits excellent performance, obtaining an error of 4.586 months in the public dataset, which is close to the performance of the current state-of-the-art methods. This method helps reduce the repetitive and mechanical work of physicians, streamline the workflow, reduce the inter-operator variation, provide a reference for clinical diagnosis, and allow physicians to focus on the accurate diagnosis of diseases.

Some deficiencies were observed. The images of different age groups in the dataset were unbalanced, which introduced some problems to the generalization of the algorithm: lack of data preprocessing and dark X-ray images with low contrast, which inevitably affected the feature learning. Some global information of the image was also disregarded; between the two BAA methods, the GP atlas is fast and focuses on the global information of the entire image, while the TW score is relatively slow and focuses on the local information of specific regions of the image. This paper mimics the process of the physician’s TW BAA method, which focuses on specific regions of the hand bones and ignores the global information.

Future work will collect additional data, balance images to reduce errors, study data preprocessing, and consider designing an attention mechanism for global–local contextual information fusion by combining the GP atlas and TW scoring methods to improve the evaluation accuracy. In addition, the multimodal data fusion of X-ray, ultrasound, and MRI of hand bones can be considered to provide new ideas for BAA. Furthermore, effectively obtaining intuitive explanations for radiologists through deep learning-based BAA methods should be investigated to help clinical diagnosis.

## Figures and Tables

**Table 1:** Bone age prediction accuracy per age group.

Age	MAE		RMSE	
(Years)	Valid	Test	Valid	Test
[0, 1)	0.046	6.554	0.046	6.554
[1, 2)	0.575	2.891	1.074	2.908
[2, 3)	3.476	2.239	5.255	2.44
[3, 4)	5.808	5.71	7.667	6.309
[4, 5)	8.761	5.04	11.08	5.492
[5, 6)	6.929	7.399	9.242	8.23
[6, 7)	7.714	7.357	9.789	8.361
[7, 8)	7.09	3.742	9.594	5.157
[8, 9)	7.137	5.024	8.934	5.842
[9, 10)	8.334	8.087	10.8	10.22
[10, 11)	7.987	4.125	10.425	4.964
[11, 12)	6.924	4.281	9.015	5.546
[12, 13)	5.668	3.545	7.671	4.551
[13, 14)	4.639	2.583	6.456	3.754
[14, 15)	3.861	4.124	5.669	4.65
[15, 16)	6.486	3.931	8.009	4.265
[16, 17)	3.659	5.06	4.593	5.577
[17, 18)	4.225	/	5.366	/
[18, 19)	1.665	20.707	3.457	20.707
[19, 20)	2.045	/	2.662	/
AVG	6.22	4.586	8.406	5.96

**Table 2:** Results of the ablation experiments in the validation and testing sets. (SG means trained separately by sex; GT means sex token).

Image	SG	GT	MAE		RMSE	
			Valid	Test	Valid	Test
Whole image	×	×	8.626	8.094	11.355	10.986
	✓	×	7.459	6.483	9.875	8.355
	×	✓	7.05	5.901	9.421	7.614
ROI	×	×	7.269	6.22	9.692	7.945
	✓	×	6.846	5.41	9.162	7.128
	×	✓	6.22	4.586	8.406	5.96

**Table 3:** Comparison of the performances in terms of the mean absolute error in months between our methods and the state-of-the-art ones on the open RSNA dataset.

References	Method	MAE (months)
Larson [36]	Improved ResNet–50	6.0
González [37]	Identity label + Inception–V3	5.47
Liu [38]	Ranked learning + VGG–U–Net + GAN	6.05
Halabi [39]	Sex + Inception v3 + Dense	4.2, 4.4, 4.5 (top3)
Iglovikov [14]	U–Net + key point detection	4.97
Koitka [40]	Faster–RCNN + sex regression network	4.56
Liu [41]	One-stage attention + age recognition network	4.38
Chen [42]	Attention + joint age distribution learning	4.4
Ours	Two-stage + alignment + sex token	4.586

## Data Availability

The RSNA Pediatric Bone Age Challenge Dataset can be downloaded from https://www.rsna.org/education/ai-resources-and-training/ai-image-challenge/RSNA-Pediatric-Bone-Age-Challenge-2017, accessed on 13 July 2017.
